# Serum Sestrin-1 Concentration Is Higher in Frail than Non-Frail Older People Living in Nursing Homes

**DOI:** 10.3390/ijerph19031079

**Published:** 2022-01-19

**Authors:** Begoña Sanz, Chloe Rezola-Pardo, Haritz Arrieta, Ana Belén Fraile-Bermúdez, Janire Alonso-Puyo, Irene Molano, Ana Rodriguez-Larrad, Jon Irazusta

**Affiliations:** 1 Department of Physiology, Faculty of Medicine and Nursing, University of the Basque Country (UPV/EHU), 489040 Leioa, Bizkaia, Spain; chloe.rezola@ehu.eus (C.R.-P.); janire.alonso29@gmail.com (J.A.-P.); ana.rodriguez@ehu.eus (A.R.-L.); jon.irazusta@ehu.eus (J.I.); 2 Biocruces Bizkaia Health Research Institute, 48903 Barakaldo, Bizkaia, Spain; 3 Department of Nursing II, Faculty of Medicine and Nursing, University of the Basque Country (UPV/EHU), 20014 Donostia-San Sebastián, Gipuzkoa, Spain; haritz.arrieta@ehu.eus; 4 Department of Nursing I, Faculty of Medicine and Nursing, University of the Basque Country (UPV/EHU), 48940 Leioa, Bizkaia, Spain; anabelen.fraile@ehu.eus; 5 Residencia Sanmarcosene, Carretera de San Marcos, s/n, 20100 Errenteria, Gipuzkoa, Spain; psikologia.sanmarkosene@gureak.com

**Keywords:** aging, biomarker, dependence, frailty, physical activity, physical function, sestrin-1

## Abstract

Given the increasing prevalence of frailty and its implications for public health, the identification of biomarkers to detect frailty is essential. Sestrin-1 is a protein with a protective role in muscle function. This study aimed to determine whether the serum sestrin-1 concentration differed between frail and non-frail populations and to investigate its association with frailty-related variables in 225 older women and men living in nursing homes (Gipuzkoa, Spain). Serum sestrin-1 concentration was measured by ELISA. Frailty, dependence, anthropometry, physical function, and physical activity were determined by validated tests and tools. The associations between sestrin-1 concentration and the other variables were determined using generalized linear models. The differences between frail and non-frail individuals were analyzed by the Mann–Whitney U-test, and receiver operating characteristic (ROC) curves were constructed to calculate the capability of sestrin-1 to detect frailty. Unexpectedly, frail individuals—according to the Fried Frailty Phenotype or the Clinical Frailty Scale—had higher serum sestrin-1 concentrations than non-frail individuals. Furthermore, the higher serum sestrin-1 concentration was associated with the increased frailty scores and dependence as well as the poorer physical function and the less physical activity. Given the contradictory results regarding serum sestrin-1 and frailty, further investigation is required to propose it as a molecular biomarker of frailty.

## 1. Introduction

Frailty is a geriatric syndrome that attracts global attention, as the population of older adults rises globally with major implications for clinical practice and public health [1]. Frailty is associated with declines in physiological reserve and function across multiple organs and systems, leading to increased vulnerability to adverse health outcomes and significantly influencing the quality of life [2,3]. Frailty may lead to dependence and has been associated with a higher risk of falls, hospitalization, and increasing of comorbidities and death [4].

Given frailty’s great impact on the overall society, together with its reversible nature, finding tools for frailty’s early detection is critical [5] to implement specific actions aimed at reducing frailty to ensure the quality of care provided to the aging [6]. Despite the existence of several validated tests and tools for the identification of frailty [4], they tend to be time-consuming [1]. Moreover, as proposed by Ferrucci et al. [7], changes in the phenotypic and functional manifestations due to molecular-level perturbations are buffered by homeostatic mechanisms. Thus, the identification of molecular biomarkers for frailty could help to reduce morbidity and mortality in older adults, as well as to improve the quality of life [8]. Frailty is frequently preceded by sarcopenia, a progressive and generalized skeletal muscle disorder characterized by the loss of muscle mass and function [9,10]. For that reason, most investigations on frailty biomarkers have focused on muscle-related molecules.

Sestrins were first discovered in 1994 as a target of the tumor suppressor p53 [11]. It has been found that members of the sestrin protein family are highly evolutionarily conserved in the animal kingdom [12], with functions related to stress response [13], antioxidant processes [14], and autophagy [15]. In this way, the role of sestrins has been associated with the lifespans in different animal models such as *Caenorhabditis elegans* [16] and *Drosophila melanogaster* [17]. Recently, some authors have proposed that in mammals, sestrins may be related not only with the lifespan, but also with the healthspan [17]. Among sestrin family members, sestrin-1 is characterized by strong expression in the skeletal muscle [12,18], and some studies have proposed that low levels of sestrin-1 may be a biomarker for sarcopenia [19] or frailty [20]. In the same vein, Segalés et al. [21] recently proposed that in murine models, sestrin-1 may protect muscles against aging-induced atrophy. However, despite the protective role in aging found for sestrin-1 by several authors [17,22], its function in decreasing T-cell-mediated immunity by senescence mechanisms has also demonstrated [23]. In exercised mice, opposite results have been found regarding sestrin-1 expression in muscle when analyzing the effect of acute or chronic exercise [24]. Thus, the role of sestrins in muscle function [24] and aging [25] remains ambiguous.

The aim of this work was to investigate the association of the serum sestrin-1 concentration with frailty, assess differences in its concentration between frail and non-frail older adults living in nursing homes and evaluate the capability of sestrin-1 as a biomarker to identify frailty in the assessed population. We also sought to understand the association of the serum sestrin-1 concentration with parameters usually linked to frailty such as the dependence in activities of daily living (ADL), anthropometry, physical function, and physical activity.

## 2. Materials and Methods

### 2.1. Study Design and Participants

This is a secondary analysis of the baseline data from three randomized controlled trials, of which the primary outcomes have been previously published [26,27,28]. The effects of a multicomponent physical exercise program [29] on people living in nursing homes were evaluated in the first trial. The effects of a multicomponent physical exercise program were compared with a dual-task intervention in the second trial [30] and a walking intervention in the third. These studies were conducted at 14 nursing homes in Gipuzkoa, Spain (ACTRN12616001044415, ACTRN12618000536268, and NCT03996083) between October 2016 and December 2018.

A total of 225 older people (157 women and 68 men) living in nursing homes were included in the present study based on the following criteria (≥70 years old, scored at ≥50 on the Barthel Index for ADL (0–100) [31], and scored at ≥20 (range: 0–35) on the MEC-35 test (a modified and validated version of the Mini Mental State Examination (MMSE) in Spanish)) [32] and could stand up and walk independently for at least 10 meters.

### 2.2. Determination of the Serum Sestrin-1 Concentration

Blood sample collection took place in the morning following an overnight fast. Thereafter, tubes were centrifuged at 5000× *g* for 10 min. The serum obtained from each participant was aliquoted and stored at −80 °C for further analysis. The serum sestrin-1 concentration was analyzed using a commercial kit (MBS3803290, MyBioSource, San Diego, CA, USA), following the manufacturer’s instructions. Fluorometric quantification was performed with a FLUOstar OPTIMA Microplate Reader (ThermoFisher Scientific, Waltham, MA, USA) and Optima Control software version 2.20 (BMG, LABTECH, Ortenberg, Germany). The samples were analyzed in duplicate, and the values were averaged.

### 2.3. Sociodemographic Data

Sociodemographic data (sex and age) were collected from the nursing home databases.

### 2.4. Frailty

Frailty status was assessed using the Fried Frailty Phenotype [2], the Clinical Frailty Scale [33], and the Tilburg Frailty Indicator [34]. Based on the Fried Frailty Phenotype, frailty was identified by the presence of three or more of the following signs/symptoms: unintended weight loss, exhaustion, weakness, slow gait speed, and low physical activity [2]. On the Clinical Frailty Scale, clinical judgment was used to determine frailty status. The possible scores ranged from 1 to 9, and participants with a score of 6 points or more were considered frail [33]. The Tilburg Frailty Indicator comprised 15 questions on physical, psychological, and social domains of frailty. Participants with a score of 5 or higher were considered frail [34]. Table 1 summarizes information about the tests employed for identifying frailty. Accredited and experienced professionals performed all the tests.

### 2.5. Dependence in ADL

The Barthel Index reflects dependence in ADL, with a score range of 0 to 100 and lower scores reflecting a higher degree of dependence. Barthel Index scores [31] were recorded from the nursing home databases.

### 2.6. Anthropometry

Waist and hip circumferences were measured with a non-elastic anthropometric tape to the nearest 0.1 cm. The body mass was measured with an Omron digital scale to the nearest 0.1 kg, and the height was measured with a Holtain stadiometer to the nearest 0.1 cm. The body mass index (BMI) and the waist-to-hip ratio were calculated based on the mass and height (kg/m^2^) and the waist and hip circumferences, respectively.

### 2.7. Physical Function

Participants’ physical function was evaluated by multiple tests. The handgrip strength was measured with a Jamar dynamometer [35], the aerobic capacity with a 6 min walk test, and a lower limb strength with the chair-stand test from the Senior Fitness Test [36]. The dynamic balance was assessed with the Timed Up and Go test [37] and a static balance with the Berg Balance Scale [38]. The functional performance of lower limbs (static balance, gait speed, and lower limb strength) was evaluated with the Short Physical Performance Battery (SPPB) [39].

### 2.8. Physical Activity

Physical activity was measured using an Actigraph GT3X model (Actigraph LLC, Pensacola, FL, USA) accelerometer. The monitor was worn on the hip with a belt for a seven-day period by the participants, who were requested to remove it only for sleeping and bathing. The recorded data files were downloaded and processed using Actilife software (version 6, Actigraph, 2012). Only days on which the monitors were worn for 10 or more hours were considered valid, and at least three days of data were required to validate the recorded data [40]. Steps per day were recorded, along with the number of minutes per day spent in intensity-specific categories. The physical activity was classified according to the classification developed by Freedson [41] as light (100–1951 counts per minute (CPM)), moderate (1952–5724 CPM), or vigorous (>5725 CPM). Moderate-to-vigorous physical activity (MVPA) was defined as the sum of minutes spent in moderate and vigorous physical activities (≥1952 CPM).

### 2.9. Statistical Analyses

The Kolmogorov–Smirnov test was used to verify the normality of quantitative variables. Continuous variables with a normal distribution such as the BMI, the waist-to-hip ratio, the handgrip, and the results of the 6 min walk test were described using the mean and the standard deviation. Continuous variables with no normal distribution (sestrin-1, the age, the body mass, the height, the chair-stand test, the Timed Up and Go test, and those corresponding to physical activity) and ordinal variables (the Fried Frailty Phenotype, the Tilburg Frailty Indicator, the Clinical Frailty Scale, the Barthel Index, the Berg Balance Scale, and the SPPB) are expressed as the median and the interquartile range. Categorical variables (sex and frailty status) are expressed as the number and the percentage (%).

The association between the serum sestrin-1 concentration and the remaining variables was determined using generalized linear models. Values and scores were introduced as dependent variables, and the sestrin-1 concentration was considered as covariable (model 1). Models were also controlled by sex, age, and BMI (model 2). The Mann–Whitney U-test was used to analyze differences in the serum sestrin-1 concentration between the frail and non-frail populations.

Receiver operating characteristic (ROC) curves were also constructed to calculate the area under the curve (AUC), considering frailty as a state variable and serum sestrin-1 concentration as a test variable. The cutoff points were calculated by the Youden Index [42], and the specificity and sensibility of each curve for identifying frailty were also determined. AUC values of >0.7, >0.8, and >0.9 were considered acceptable, excellent, and outstanding, respectively.

A *p*-value of <0.05 was considered significant. All statistical analyses were performed using SPSS software v.26 (IBM, Chicago, IL, USA).

## 3. Results

### 3.1. Descriptive Characteristics of the Sample

Participants’ descriptive data are shown in Table 2. The median of the serum sestrin-1 concentration was 10.25 ng/mL (4.6–11.65). The median of the age of the 225 participants was 84.8 years (78.7–91.5); 157 (69.8%) were women and 68 (30.2%) were men.

### 3.2. Associations of the Serum Sestrin-1 Concentration with the Rest of the Variables

The associations between the serum sestrin-1 concentration and all the analyzed variables were similar in the generalized linear models (Table 3) without controlling (model 1) and when controlling by sex and age (model 2). No association was found with age or with anthropometry data.

When frailty scores were analyzed (without controlling; controlled by sex, BMI and age), The Fried Frailty Phenotype (β = 0.033, W = 4.314, *p* = 0.038; β = 0.034, W = 4.671, *p* = 0.031) and the Clinical Frailty Scale (β = 0.131, W = 40.789, *p* < 0.001; β = 0.133, W = 42.430, *p* < 0.001) were positively associated with the serum sestrin-1 concentration. No association was found between the serum sestrin-1 concentration and the Tilburg Frailty Indicator score (*p* > 0.05). The serum sestrin-1 concentration was negatively associated with the Barthel Index, reflecting the association of the higher serum sestrin-1 concentration with more dependence in ADL (β = −0.619, W = 7.620, *p* = 0.006; β = −0.647, W = 8.684, *p* = 0.003).

Regarding physical function, no association was found between the serum sestrin-1 concentration and the handgrip. Negative associations of the serum sestrin-1 concentration were found with the 6-min walk test (β = −5.513, W = 12.357, *p* < 0.001; β = −5.649, W = 15.209, *p* < 0.001), the chair-stand test (β = −0.129, W = 4.156, *p* = 0.041; β = −0.125, W = 3.975, *p* = 0.046), the Timed Up and Go test (β = −0.009, W = 18.114, *p* < 0.001; β = −0.009, W = 19.650, *p* < 0.001), the Berg Balance Scale (β = −0.296, W = 4.822, *p* = 0.028; β = −0.310, W = 5.784, *p* = 0.016), and the SPPB (β= −0.143, W = 10.556, *p* < 0.001; β= −0.146, W = 11.762, *p* < 0.001).

Negative associations were also found, when the serum sestrin-1 concentration was analyzed with physical activity related variables such as steps/day (β = −42.143, W = 4.581, *p* = 0.032; β = −41.940, W = 5.246, *p* = 0.022) and time spent in light physical activities (β = −2.140, W = 4.988, *p* = 0.026; β = −2.018, W = 4.821, *p* = 0.028) or in light-moderate-to-vigorous physical activities (β = −2.207, W = 5.105, *p* = 0.024; β = −2.084, W = 4.972, *p* = 0.026).

### 3.3. Serum Sestrin-1 Concentration in Frail and Non-Frail Populations

**Figure 1:**
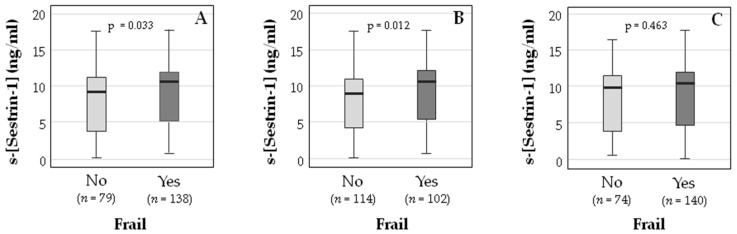
Medians and interquartile ranges of the serum sestrin-1 concentration (s-[Sestrin-1] (ng/mL)) in frail and non-frail participants following the Fried Frailty Phenotype (**A**), the Clinical Frailty Scale (**B**), and the Tilburg Frailty Indicator (**C**) criteria.

When the serum sestrin-1 concentration was compared between the frail and non-frail populations (Figure 1), it was higher in the frail group when assessing frailty with the Fried Frailty Phenotype (9.2 (3.7–11.25) vs. 10.57 (5.20–11.91) ng/mL, *p* = 0.033) and the Clinical Frailty Scale (9.02 (4.28–11.10) vs. 10.7 (5.6–12.21) ng/mL, *p* = 0.012), whereas no significant difference was found in the case of the Tilburg Frailty Indicator (9.82 (3.92–11.5) vs. 10.4 (4.63–11.94) ng/mL, *p* = 0.463).

### 3.4. ROC Curves

**Figure 2:**
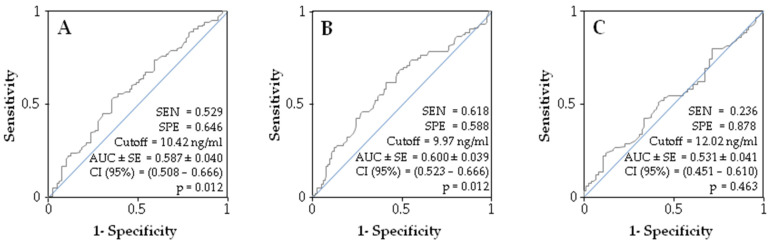
Receiver operating characteristic (ROC) curves for the serum sestrin-1 concentration as a test variable and frailty following the Fried Frailty Phenotype (**A**), the Clinical Frailty Scale (**B**), and the Tilburg Frailty Indicator (**C**) criteria as state variables. SEN, sensitivity; SPE, specificity; AUC ± SE, area under the curve ± standard error; CI, confidence interval.

ROC curves were plotted to illustrate the ability of sestrin-1 to identify frailty (Figure 2). When frailty was determined by the Fried Frailty Phenotype (Figure 2A), sestrin-1’s ability to identify frailty was significant (*p* = 0.033) with a cutoff value of 10.42 ng/mL. Despite these significant results, the AUC (0.587) was too low to be considered acceptable; the sensitivity and the specificity were 0.529 and 0.646, respectively. Similar results were obtained, when frailty was determined by the Clinical Frailty Scale (Figure 2B), where despite its significance (*p* = 0.012) and cutoff value of 9.97 ng/mL the AUC (0.600) was too low to be considered acceptable, the sensitivity and the specificity were 0.618 and 0.588, respectively. In the case of the Tilburg Frailty Indicator (Figure 2C), sestrin-1’s capability of identifying frailty was not significant (AUC = 0.531, *p* = 0.463; sensitivity = 0.236, specificity = 0.878).

## 4. Discussion

We have analyzed the serum sestrin-1 concentration in a population of older people living in nursing homes. No association was found between sestrin-1 and the sociodemographic or anthropometric parameters. However, the serum sestrin-1 concentration was positively associated with scores of the Fried Frailty Phenotype and Clinical Frailty Scale frailty indicators and was negatively associated with Barthel Index, physical function, and physical activity. Furthermore, frail individuals—according to the Fried Frailty Phenotype and the Clinical Frailty Scale—had higher serum sestrin-1 concentrations than non-frail participants. Although ROCs showed significance for the prediction of frailty following the Fried Frailty Phenotype and Clinical Frailty Scale criteria, their ability for predicting frailty was low.

Today, most published studies related to sestrins have been developed in invertebrate animal models that express only one sestrin gene (Sesn) [13], such as *Caenorhabditis. elegans* [16] and *Drosophila melanogaster* [17]. Mammals express a set of conserved proteins (sestrin-1, sestrin-2, and sestrin-3) [12]. In both invertebrate and mice animal models, genetic expression, as well as protein expression and/or the concentration of sestrins, has been positively associated with better health parameters as well as the healthy aging and lifespan [16,17,21]. Studies carried out in humans in relation to sestrin-1, although scarce, also show a negative association of this protein with parameters related to frailty. For example, Rai et al. [20] have found a higher serum sestrin-1 concentration in older non-frail people than in frail people. In the same line, Rajan et al. [19] recently proposed that sestrin-1 may be suggested as a sarcopenia biomarker, given that this protein was found in a higher concentration in people without sarcopenia than in those with sarcopenia. In the two studies cited, participants were community-dwelling Indians aged around 76 and 70 years old, respectively, and with BMIs of 23 and 24 kg/m^2^, respectively.

The results of our study results are precisely the opposite of those mentioned in the previous paragraph. That is, the serum sestrin-1 concentration was higher in frail participants following the Fried Frailty Phenotype and the Clinical Frailty Scale and in those with more dependence in ADL, reflecting a worse physical function and a lower physical activity. Despite these seemingly contrary results, our results agreed with some authors who proposed that the prolonged activation of sestrins under chronic stress may cause negative effects for the organism [25]. Taking into account that participants in our study were older (median age: 84.8 (78.7–91.5)) and had a higher BMI (28.8 ± 5 kg/m^2^) than participants in previous studies, these differences between our results and others’ may be due to the higher rate of chronic stress accumulated by the participants in our study. This idea can be supported considering that in older people, physical degeneration and illnesses accompanying aging may amplify the stress response [43] and the increased BMI per se can also lead to increased chronic stress [44]. These different results may also be due to the considerable variation reported in values of BMI and physical function among Asian and Caucasian populations [45]. Otherwise, there was no association of sestrin-1, nor any significant difference between frail and non-frail people following the Tilburg Frailty Indicator, which measures not only aspects related to physical frailty, but also its psychological and social aspects [34]. Sestrin-1’s role is linked to muscle [17], so this result could be expected in our analysis.

In one study developed using blood samples from young and old independent volunteers, as well as mice models, Lanna et al. [23] have found that, in contrast to their well-documented anti-aging properties, sestrins induced multiple characteristics of senescence in T-cells and their expression is elevated in CD4+ T-cells. In exercised mice, Crisol et al. [24] have reported that sestrin-1 expression in muscle was increased when acute exercise was performed, whereas chronic exercise provoked its diminution. Likewise, contradictory results have been described for sestrin-2 and tumor pathology, since bidirectional functions (as tumor-suppressing and oncogene) have been found for this protein in various cancer types [46].

A similar controversy has also surrounded other molecules related to muscle function that have been proposed as biomarkers for frailty such as myostatin [47,48,49,50,51], follistatin [48,50,51,52], and renin–angiotensin system elements [53,54,55,56,57]. Some authors have proposed that myostatin may act as a chalone, by turns restraining skeletal muscle growth in response to unfavorable metabolic scenarios or decreasing its own activity, when there is no need to restrain growth [50,51,58,59,60]. The seemingly contradictory results between the protective role assigned to sestrin-1 in muscle physiology and its positive association with frailty found in the present work potentially suggest that sestrin-1 may also have a function in regulating muscle growth and function in unfavorable scenarios. This hypothesis would be in agreement with the role of sestrins as central integrators of anabolic and degradative pathways [21].

Sestrin-1 is a known regulator of mammalian target of rapamycin complex 1 (mTORC1) [61,62]; myostatin [63], follistatin [64], and renin–angiotensin system elements [65] are also related to mammalian target of rapamycin (mTOR), although in different ways. mTOR is well defined as being key to the regulation of aging-associated cell metabolism [66,67], with a pivotal role in the anabolic and catabolic signaling of skeletal muscle [66]. Recently, Picca et al. [68] have described a U-shaped association between mTOR and cognitive function along the aging process. Thus, it may be that the seemingly contradictory results regarding certain putative molecular biomarkers of frailty are due to the up-regulation of compensatory systems on the frail organism, resulting in a U-shaped association; that is, during aging process, the serum concentration of such molecules is affected in one sense (increasing or decreasing), but later, the organism’s compensatory mechanisms provoke the opposite effects (decreasing or increasing, respectively) [47,48,50,51,52,53,54,55,56,57]. This is merely a general hypothesis that must to be confirmed for each molecule proposed as a biomarker. Research into the discovery of molecular biomarkers must have a real impact on clinical practice and therefore on public health policies, but it appears indispensable to develop assays at the molecular level that facilitate a thorough understanding of the pathways of regulation for each of these molecules. What is more, because multiple molecular pathways are involved in the aging process and can all contribute to various aspects of frailty, a panel of valid biomarkers in combination with measures of frailty would facilitate both diagnosis and follow-ups in preclinical and clinical settings [69].

This work has several limitations. The study was carried out using a sample of people living in nursing homes; thus, our results cannot be extrapolated to populations that do not satisfy the inclusion criteria of this study. Comparison of our results with those obtained in younger and less frail populations would have helped us to obtain more robust conclusions. In addition, given that sestrin-1’s function is closely linked to the muscle, to reach more robust conclusions, it would have been useful to have data related to participants’ body composition and sarcopenia status. The study aimed to analyze the putative role of circulating sestrin-1 in frailty and its association with the remaining determined parameters. However, more information about the role of sestrin-1 in ageing could have been obtained, if we had analyzed muscle biopsies. Despite its limitations, this work also contains several strengths that must be taken into account. For example, despite the general awareness of sestrin-1’s role in aging, to the best of the authors’ knowledge, only two works have studied its association with frailty or sarcopenia in humans, and this is the first to analyze sestrin-1 in people living in nursing homes and its association with physical function, physical activity, and dependence, as well as frailty following three different scales and indices.

## 5. Conclusions

In this population of older people living in nursing homes, the higher serum sestrin-1 concentration was associated with the increased frailty and dependence, as well as with the poorer physical function and the lower physical activity. Even though published research about the association of sestrin-1 with frailty and frailty-related outcomes is scarce, our results appear to contradict the extant literature. Sestrin-1 is synthesized in the skeletal muscle and is associated with aging-related parameters. However, more research must be developed to increase knowledge about sestrin-1 molecular regulation and its role in frailty. These investigations should be approached from a multidisciplinary perspective, including research into other sestrin-1-related molecules and studies of the implications that such knowledge has for public health. All of these research avenues have the potential to improve the quality of frailty care provided to older people.

## Figures and Tables

**Table 1:** Summary information about the Fried Frailty phenotype, the Clinical Frailty Scale, and the Tilburg Frailty Indicator.

Test	Assessed variables	Score	
		Range	cutoff
Fried Frailty Phenotype [2]	Unintentional weight loss Low handgrip strength Exhaustion Slow gait speed Low physical activity	0–5	≥3
Clinical Frailty Scale [33]	Frailty status based on clinical judgment, from very fit to terminally ill	1–9	≥6
Tilburg Frailty Indicator [34]	Physical domain (8 items) Psychological domain (4 items) Social domain (4 items)	0–15	≥5

**Table 2:** Descriptive data of the analyzed variables. Quantitative non-normal variables and ordinal variables are expressed as the median and the interquartile range (IQR). Categorical variables are shown as *n* and %. Quantitative normal variables are shown as the mean ± the standard deviation (SD).

**Variables**	**N**		
Sestrin-1 (ng/mL), median (IQR)	225	10.25	(4.6–11.65)
Sociodemographic data			
Age (years), median (IQR)	225	84.8	(78.7–91.5)
Sex			
Female, n (%)		157	(69.8)
Male, n (%)		68	(30.2)
Frailty			
Fried Frailty Phenotype (score: 0–5), median (IQR)	217	3	(2–4)
Non-frail (score: <3), n (%)		79	(36.4)
Frail (score: ≥3), n (%)		138	(63.6)
Clinical Frailty Scale (score: 1–9), median (IQR)	216	5	(3–6)
Non-frail (score: <6), n (%)		114	(52.8)
Frail (score: ≥6), n (%)		102	(47.2)
Tilburg Frailty Indicator (score: 0–15), median (IQR)	214	6	(3.75–8)
Non-frail (score: <5), n (%)		74	(34.6)
Frail (score: ≥5), n (%)		140	(65.4)
Dependence in activities of daily living			
Barthel Index (score: 0–100), median (IQR)	223	85	(70–93)
Anthropometry			
Body mass (kg), median (IQR)	222	66.1	(58.7–76.4)
Height (m), median (IQR)	222	1.51	(1.47–1.58)
Body mass index (kg/m^2^), mean ± SD	222	28.8	±5.0
Waist-to-hip ratio, mean ± SD	222	0.98	±0.07
Physical function			
Handgrip (kg), mean ± SD	225	18.0	±7.8
6-min walk test (m), mean ± SD	224	245.7	±105.3
Chair-stand test (n/30 s), median (IQR)	224	7	(3–10)
Timed Up and Go test (m/s), median (IQR)	223	0.33	(0.23–0.46)
Berg Balance Scale (score: 0–56), median (IQR)	221	47	(39.5–51.5)
Short Physical Performance Battery (score: 0–12), median (IQR)	221	6	(4–9)
Physical activity			
Steps (n/day), median (IQR)	209	854	(424–1509)
Light physical activity (min/day), median (IQR)	209	87.1	(49.1–125.8)
Moderate-to-vigorous physical activity (min/day), median (IQR)	209	0.57	(0.29–1.14)
Light-moderate-to-vigorous physical activity (min/day), median (IQR)	209	89.1	(49.8–127.2)

**Table 3:** Generalized linear models for the analyzed variables as dependent variables. Model 1: for the serum sestrin-1 concentration as a predictor variable. Model 2: for the serum sestrin-1 concentration as a predictor variable, age and BMI were considered as covariables, and sex was considered as a factor. In the case of anthropometry variables, BMI was not used as a covariable (**).

		Model 1			Model 2		
Dependent Variable	N	β	Wald’s	*p*	β	Wald’s	*p*
			χ^2^			χ^2^	
Sociodemographic data							
Age (years)	225	0.060	0.003	0.957	−0.019	0.042	0.838
Frailty							
Fried Frailty Phenotype (score: 0–5)	217	0.033	4.314	0.038	0.034	4.671	0.031
Clinical Frailty Scale (score: 1–9)	216	0.131	40.789	<0.001	0.133	42.430	<0.001
Tilburg Frailty Indicator (score: 0–11)	214	0.032	0.482	0.488	0.033	0.562	0.454
Dependence in activities of daily living							
Barthel Index (score: 0–100)	223	−0.619	7.620	0.006	−0.647	8.684	0.003
Anthropometry (**)							
Body mass (kg)	222	−0.105	0.246	0.620	−0.083	0.179	0.672
Height (m)	222	<0.001	0.074	0.786	<0.001	0.001	0.981
Body mass index (kg/m^2^)	222	−0.038	0.245	0.621	−0.042	0.313	0.576
Waist-to-hip ratio	222	<0.001	0.010	0.921	<0.001	0.031	0.861
Physical function							
Handgrip (kg)	225	−0.001	<0.001	0.933	0.026	0.110	0.740
6-min walk test (m)	224	−5.513	12.357	<0.001	−5.649	15.209	<0.001
Chair-stand test (n/30 s)	224	−0.129	4.156	0.041	−0.125	3.975	0.046
Timed Up and Go test (m/s)	223	−0.009	18.114	<0.001	−0.009	19.650	<0.001
Berg Balance Scale (score: 0–56)	221	−0.296	4.822	0.028	−0.310	5.784	0.016
Short Physical Performance Battery (score: 0–12)	221	−0.143	10.556	<0.001	−0.146	11.762	0.001
Physical activity							
Steps (n/day)	209	−42.143	4.581	0.032	−41.940	5.246	0.022
Light physical activity (min/day)	209	−2.14	4.988	0.026	−2.018	4.821	0.028
Moderate-to-vigorous physical activity (min/day)	209	−0.067	2.121	0.145	−0.068	2.245	0.134
Light-moderate-to-vigorous physical activity (min/day)	209	−2.207	5.105	0.024	−2.084	4.972	0.026

## Data Availability

The data that support the findings of this study are available from the corresponding author, B.S., upon reasonable request.
